# Implementation of good manufacturing practices (GMP) to improve the quality of smoked fish (*Scombercolias*)

**DOI:** 10.1016/j.heliyon.2024.e27401

**Published:** 2024-03-03

**Authors:** Vivianne Geraldo, Jerry Ampofo-Asiama, Cynthia A. Adinortey, Isaac Okyere, Samuel Kofi Tulashie, Alexander Tetteh Kwasi Nuer, Samuel Bridge Nkansah, Selorm Omega, Salifu Seidu-Larry, Nazir Kizzie-Hayford

**Affiliations:** a Department of Biochemistry, University of Cape Coast, Ghana; b Department of Molecular Biology and Biotechnology, University of Cape Coast, Ghana; c Centre for Coastal Management (Africa Centre of Excellence in Coastal Resilience), University of Cape Coast, Ghana; d Department of Chemistry, University of Cape Coast, Ghana; e Department of Agricultural Economics and Extension, Ghana

**Keywords:** Smoked fish, Good manufacturing practices (GMP), Microbiological quality

## Abstract

For several years, fish smoking has been the widely adopted processing method among artisanal fish smokers located along the coastal zones in many parts of West Africa including Ghana. However, several issues pertaining to biochemical and microbiological contaminants still remain, mainly because of the suboptimal, unhygienic fish handling during the processing. To help curtail the problem, we developed and implemented a simple good manufacturing practice (GMP) system for experimentation at two local fish smoking facilities (Facility A, F_A_; Facility B, F_B_) to assess the effectiveness for improving the quality of smoked fish. The implementation of GMP did not affect the physical properties of the smoked fish but improved the peroxide value, total volatile base nitrogen, polyaromantic hydrocarbons and histamine levels. The total aerobic counts decreased from 3.96 ± 0.12 cfu/g to 1.52 ± 0.28 cfu/g (F_A_) or from 4.10 ± 0.2 cfu/g to 1.85 ± 0.85 cfu/g, (F_B_). The coliforms and *Escherichia coli* decreased respectively from 1.69 ± 0.12 cfu/g and 1.15 ± 0.21 cfu/g (F_A_) and from 1.74 ± 0.37 cfu/g and 1.24 ± 0.37 cfu/g, (F_B_) to below detection (no observed colony) after introducing the single use of potable water, use of smoking oven and fish core temperature of 108.1 ± 7.5 °C and 82.5 ± 3.9 °C, respectively for 2 h, wearing of safety apparels, drying and cooling of smoked fish under nets, and the use of waste disposal bins. The results show that sensitization and training of fish smokers in GMP may be relevant for improving the microbial and overall quality of smoked fish.

## 1 Introduction

Fish and fish products are considered to be one of the healthiest protein sources accounting for approximately 17 % of animal protein intake with increasing global demand [1]. In Ghana, the level of fish consumption is approximately 25 kg/annum per capita, which is higher than 10.5 kg/annum per capita and 18.9 kg/annum per capita for Africa and the World, respectively [2]. This shows that fish is an important food commodity in Ghana. Again, Ghana imports over 191.000 metric tons of fish annually, consisting of mainly mackerel and Sardine, meeting only about 50 % of the national fish demand [3].

Preservation methods such as freezing, drying, salting, and smoking are mainly employed to reduce post-harvest losses of fish [4]. Fish smoking is one of the oldest processing methods, being widely preferred to other methods because of the characteristic smoky flavor and colour. In addition, smoked fish is sometimes considered a “ready-to-eat” product because either the whole or powdered smoked fish is added to finished meals [4,5]. Hot fish smoking involves an appropriate combination of temperature and time (at least 70 °C for 30 min) sufficient to cause the complete coagulation of fish proteins, killing of parasites and the destruction of non-sporulating pathogens. The smoking process is known to injure spores of human health concern [6] providing the required killing of microbes, cooking and drying effects, which further inhibits the growth of bacteria [7,8]. Despite the anti-microbial effects of smoking on fish, safety issues, mainly emanating from poor handling and environmental hygiene remain insufficiently addressed in Ghana [9]. Many of the problems can be traced to the low or lack of formal education or limited training/sensitization programs on food safety among fish processors [7,9]. To help improve the quality and safety of smoked fish, it is expedient to develop and implement food hygiene and safety protocols for fish smoking by examining the whole processing chain from raw material acquisition to the final product packaging stage. Food safety hazards from smoked fish originate mainly from biological sources such as pathogenic bacteria including *Listeria monocytogenes*, *Escherichia coli*, *Salmonella* spp., *Vibrio* spp., and *Shigella* spp. Thermotolerant bacterial spores from i. e, *Clostridium* spp., and *Bacillus* spp., may survive in fish products even after prolonged smoking at high temperatures [10,11]. Biogenic amines and toxic compounds such as polycyclic aromatic hydrocarbons (PAH) may also occur [10]. The presence of food hygiene indicators such as *E. coli* and *Salmonella* spp. raises concerns with regards to the proper handling of fish after smoking [[12], [13], [14]]. In addition, PAH which shows a high carcinogenic potential has been reported to occur above the safe levels of 2 μg/kg for benzo[*a*]pyrene, being in the range of 510.59 μg/kg – 1461.79 μg/kg in Ghanaian smoked fish [15,16]. Traditional small-scale fish processing is dominated by women, especially in tropical African regions. The high operational cost, lack of financial support and the lack or low levels of education are among the factors that impede the adaptation of food safety practices by small to medium fish processors [17,18]. As a result, focusing on the basic requirements of GMP with the aim of simplifying its adoption, could be a good option for small-medium scale fish processors to integrate the practices into fish processing, and hence, improve on the fish quality. The application of a basic GMP, which could be used as a preventive approach to quality assurance, can allow the improvement in safety handling of fish during smoking to minimize or prevent the incidence of food hazards [19,20].

Therefore, in this study, the processing chain for fish smoking was scrutinized and assessed for physicochemical, microbial, and chemical quality. Then, a basic GMP system was developed and implemented, and the chain re-evaluated to assess the effects on the quality of smoked fish.

## 2 Materials and methods

### 2.1 Study location and sampling

Logistical limitations permitted the random recruitment of two fish smoking facilities (Facility A (F_A_), Brofoyedru, GPS: CC-024-0046 and Facility B (F_B_), Duakor, GPS: CC-195-0961), both of which are local communities located in the Cape Coast Metropolis in the Central Region of Ghana for the study. The objective of the study was explained to the operators before the study was undertaken. Both operators were women who had more than 15 years’ experience of fish smoking. Fish smoking was usually done on a micro-scale basis involving two to three workers per facility with a production volume of approx. 150 kg per week, depending on the fishing season.

### 2.2 The fish smoking process

**Fig. 1:**
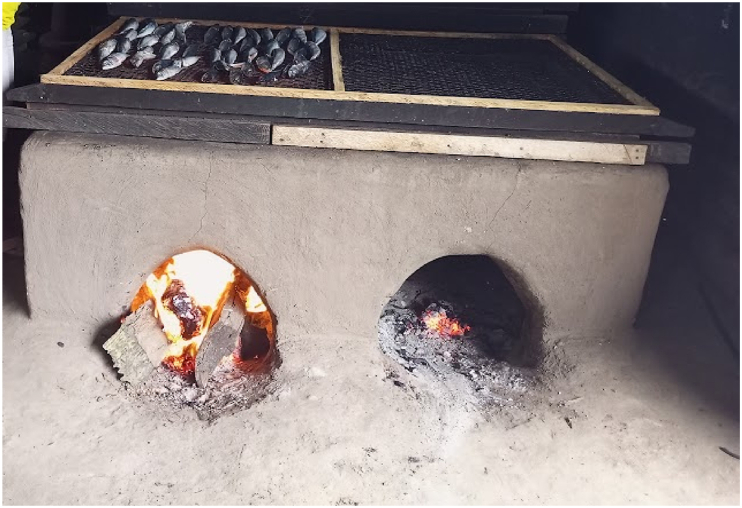
A traditional Chorkor oven.

To assess the effect of the GMP application on the quality of smoked fish, the operators were allowed to apply their pre-existing knowledge for processing smoked fish without any interventions. The research team studied the entire process chain from the raw material (unsmoked fish) acquisition to the packaging stages of the smoked fish. This was done so as to allow for the documentation of the fish smoking process. Atlantic chub mackerel (*Scomber colias*) was used for smoking because of the availability and low cost. Additionally, the more commonly used Chorkor oven, which is a rectangular brick equipped with two low frontal openings for fuelling firewood and a flat top covered with smoking plates made up of wire mesh set in a wooden framework (Fig. 1) [21,22] was used for the study. The first phase of fish smoking operations at the processing facilities, F_A_ and F_B_ were observed on the 18th and March 25, 2022, respectively. Subsequently, similarities and differences in the smoking process at the two facilities were noted and harmonized to obtain a single fish smoking operation in which the basic GMP could be applied. Then the second phase of the fish smoking operation was conducted on 15th and 22nd June 2022 at both F_A_ and F_B_, respectively. To compare the impact of the GMP intervention, the same lot of fish was used at the facility F_A_, whilst because of proximity, a different source but same species and same lot was used at facility F_B_ for the first and second phases of the study. The smoked fish samples from both facilities were transported on ice in sterile zip-lock plastic bags to the Laboratory of the Department of Biochemistry, University of Cape Coast and stored in a refrigerator at 4–6 °C. Microbiological analyses was done within 24 h of storage.

### 2.3 Fish quality analyses

Microbiological and biochemical analyses were carried out on fish samples produced before and after implementing the GMP to determine the impact on fish quality.

#### 2.3.1 2.3.2 microbiological analyses

For the microbiological analysis, fish samples were prepared by homogenizing 10 g of smoked fish muscle in 135 mL sterile phosphate buffer saline (PBS) for 60 s. To be able to monitor the microbiological effect of the GMP intervention, only a limited group of indicator microorganisms as described by Oyelese and Oyedokun [23] were selected to include the total aerobic plate count, *E. coli*, coliforms, and fungi (yeasts and molds) and were enumerated as follows: The total aerobic bacterial count, which is an indicator of hygiene and safety [24], was determined by pour plating on plate count media (PCA, Oxoid code CM 325) and incubating at 37 °C for 24 h as described by the AOAC methods [25]. Molten Violet Red Bile Glucose (VRBG, Oxoid code CM 485) agar and Eosin Methylene Blue (EMB, Oxoid code CM 069) agar, respectively, were used for coliforms and *E. coli* enumeration and incubation at 37 °C for 24 h according to the procedure described by Corry et al. [26]. Fungi was enumerated based on the procedure by Corry et al. [26] using Sabourad Dextrose Agar (SDA, Oxoid code CM 041), and was modified by adding 1 mg/mL chloramphenicol. After pour plating, the media was incubated for 5 days at 28 °C. The microbial loads were reported as log colony forming units (log CFU/g of fish sample). Microbiological analyses of the raw and smoked fish samples were performed in triplicate.

#### 2.3.2 2.3.1 physicochemical analysis

Physicochemical analyses done for smoked fish were the moisture, pH, hardness, colour, peroxide value, total volatile base nitrogen (TVB-N), histamine levels and PAH composition. The moisture content was determined as described by the AOAC [27] methods by drying the comminuted fish samples in an oven at 105 °C for 6 h and computing the loss on moisture as a percentage of the mass of smoked fish. The pH of the samples was determined using a pH meter (Inolab pH730) after homogenizing 10 g fish muscle in 50 mL distilled water. The hardness of the fish muscle was measured after filleting and removing the skin. Measurement was obtained from three points (back, belly and tail) along each fish fillet using a GY-4 digital penetrometer as described by Dadzie et al. [28]. The colour of the anterior, mid and dorsal sections of smoked fish was measured using CHN SPEC CS-10 colorimeter. The measured colour indices were used to estimate the browning index as explained previously [29]. All laboratory measurements were done in quadruplicates. Peroxide value of the fish oil, which was extracted using petroleum ether by the Soxhlet extraction method, was measured according to the AOAC [27] methods. Fish oil (5 g) was weighed into a 250 mL conical flask and mixed with a 30 mL acetic acid to chloroform ratio of 3:2. After adding 0.5 mL saturated potassium iodide, the solution was incubated in the dark with shaking for 1 min and diluted with 30 mL distilled water. Subsequently, the solution was titrated with 0.01 M sodium thiosulphate (Na_2_S_2_O_3_) until a pale yellow was developed. Exactly 0.5 mL of 1.0 % starch solution was added as indicator and continuously titrated until the disappearance of the blue complex. Appropriate negative controls were prepared without oil, which served as the blank. The peroxide value was estimated and expressed as milli equivalent peroxide oxygen per kg (meq/kg) sample. The method described by Socaciu et al. [30] was used for determining the total volatile basic nitrogen (TVB-N). Briefly, to 10 g of homogenized smoked fish, 90 mL of 0.6 M perchloric acid was added and centrifuged at 3000×*g* for 10 min, after which 50 mL of 30 % NaOH (30 %) was added to 20 mL of the supernatant and distilled. The distillate was transferred into a flask containing 50 mL boric acid, to which was added mixed indicators of methyl red and bromocresol green. The solution was then titrated against 0.01 M HCl, along with a blank titration. TVB-N was expressed as mg Nitrogen/Kg fish muscle. Histamine content was analyzed by homogenizing 10 g of smoked fish in 2.5% trichloroacetic acid (100 mL) using a waring blender for 2 min, filtering the suspension and removing interfering compounds on an ion-exchange chromatographic column as described by Hardy & Smith [31]. After eluting the absorbed histamine with 0.2 N hydrochloric acid, one mL of the eluate was reacted with 15 mL, 5 % sodium carbonate followed by the addition of 2 mL diazonium salt, which was allowed to react until equilibration for 10 min. Then the absorbance of the histamine complex was measured at 495 nm using a spectrophotometer (Genesys 20, Manlo Park, CA, USA). Distilled water was used as a reference. Standard histamine solutions that was similarly diazotized was used as the calibration standard [31] and the values reported as mg/kg histamine. For estimating the polyaromatic hydrocarbon content (PAH), the method described by Asamoah et al. [32] was used. Briefly, 3 g fish was homogenized in 15 mL acetonitrile, and the suspension centrifuged at 3000×*g* for 5 min. The supernatant (6 mL) was transferred into a centrifuge tube containing 150 mg of a Primary Secondary Amine (PSA), 150 mg C18 and 900 mg magnesium sulphate, and centrifuged for 5 min at 3000×*g*. Using a rotary evaporator, the supernatant (4 mL) was concentrated into a paste and re-dissolved in 1 mL acetyl acetate for gas chromatography-mass spectrometry (GC-MS) analysis. The GC-MS system consisted of Agilent 7890 B (Agilent Technologies, Santa Clara, CA, USA) in a splitless mode. After injecting 1 mL of sample at 280 °C, the oven temperature was adjusted to 70 °C for 2 min after which the temperature was allowed to rise to 150 °C at 25 °C/min, then to 200 °C at 3 °C/min and finally to 280 °C/min at 8 °C/min for 12 min. Helium served as the carrier gas at 2.25 mL/min constant flow and PAH quantification was carried out by a Clarity-GC interfaced software.

### 2.4 Data analysis

The effect of implementing the GMP on the microbiological and physicochemical quality of smoked fish was analyzed using the analysis of variance tool (ANOVA). For this, SPPS (IBM, SPSS Statistics 20) was used. All statements on significance refer to *p* < 0.05.

## 3 Results and discussion

### 3.1 Observations on the fish smoking process

**Fig. 2:**
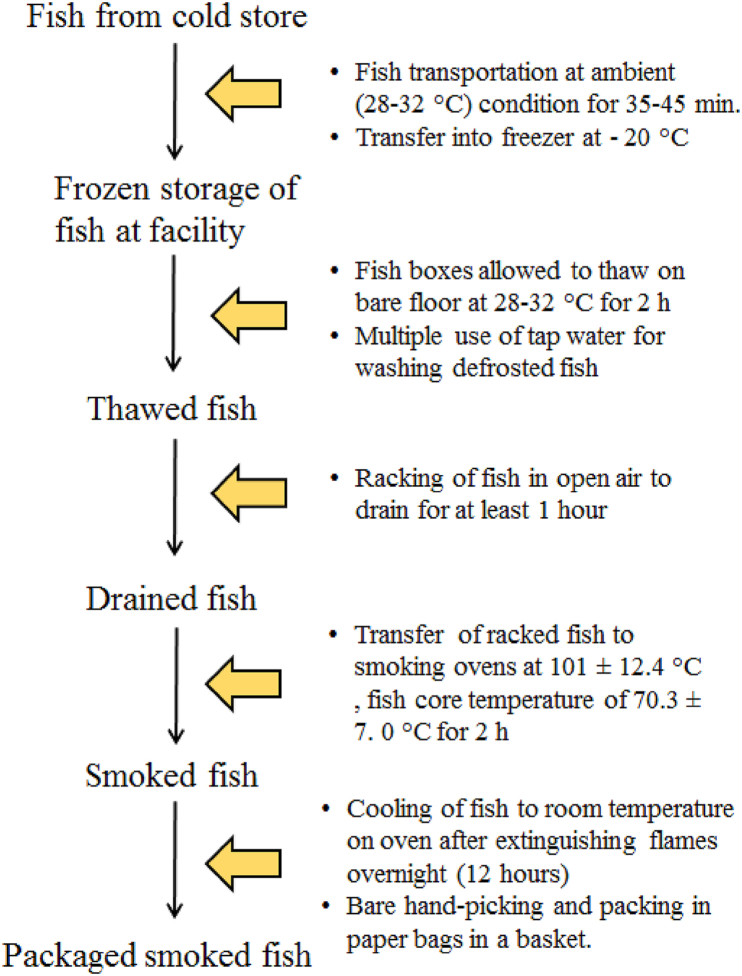
The traditional fish smoking process showing the conditions of operation.

Gap analysis conducted at the fish smoking facilities revealed that both F_A_ and F_B_ lacked the basic facilities to guarantee a safe, smoked fish quality. For example, akin to most traditional fish smoking operations in Ghana, both facilities operated under open sheds, which were typically dusty and lacked the recommended waste disposal systems. These conditions could predispose smoked fish to contaminants before, during and after processing. Monitoring of the fish processing steps (Fig. 2) used by the fish smokers revealed that after purchasing the frozen fish (mackerel, *Scomber colias*), which was packed in 20 kg blocks, wrapped in a polyethylene film and stored in paper boxes at −20 °C, they were transported under ambient conditions (28–32 °C) within 35–45 min depending on the proximity to the respective processing facilities. The Fish was allowed to thaw on the bare ground to room temperature, which took approximately 2 h. Subsequently, the fish was washed with tap water, which was used multiple times for removing blood, slime and foreign materials. Then, the washed fish was racked to drain the moisture in the open sun by Facility A (F_A_) or over a smouldering oven by Facility B (F_B_) for about 1 h. Thereafter, the drained fish was transferred into a locally constructed Chorkor oven and hot-smoked using hardwood from mango tree (*Mangifera indica*) at an external oven temperatures and fish core temperatures of 101.4 ± 13.8 °C and 71.0 ± 7.1 °C for F_A_, or from Essia wood (*Petersianthus macrocarpus*) at 100.6 ± 9.8 °C and 69.5 ± 6.2 °C for F_B_, respectively, for 2 h until sufficient cooking was achieved. The fish smoking operators left the smoked fish to cool to room temperature overnight (approx. 12 h) before picking by hand and packing into brown paper bags in a basket. Hot smoking was the preferred method of fish smoking by the operators and the smoking intensity was casually modulated by adjusting the quantity of firewood until the desired fish cooking weight, moisture, colour and aroma was obtained [4,33]. The source of fish used for smoking was determined by the fish availability, with freshly harvested fish being preferred to frozen fish because of the low cost, especially during the bumper season. However, in the lean season, the fish smoking operators relied on frozen fish from cold storage facilities [4,33] because of the limited seasonal supply.

### 3.2 Implementation of GMP

In view of the limitations in the hygienic operations of the two processing facilities, some interventions (Table 1) that could help to improve the safety of smoked fish at a minimal cost was implemented based on the potential hazards that could occur during traditional fish smoking outlined in Table 2. The cost-effective interventions were experimented in this study with the aim of helping its adoption among the operators of the local fish smoking facilities. Firstly, a cold chain was maintained by keeping the fish on ice using ice chests during the transportation to the facility. Then, the fish was thawed in bowls containing potable tap water, which was singly used for defrosting fish at 20 °C for 45 min, helping to reduce the time of exposure to micro-organisms, dirt and other contaminants. Subsequently, fish was racked under nets to prevent contact with flies and transferred to warm ovens (approx. 50 °C) to enhance dehydration for 30 min before smoking at 108.1 ± 7.5 °C, fish core temperature of 82.5 ± 3.9 °C for 2 h. The reduction in the exposure of fish to ambient temperature and the draining time could help to decrease the microbial activity, which can affect the histamine and free nitrogen and ammonia levels [34] whilst the careful adjustment of fire wood to decrease the variation in heat supply could lead to adequate cooking, smoke formation and inactivation of microorganisms, leading to chemical and microbiologically safer smoked fish products. To reduce the chance of fish recontamination, the duration for cooling of the smoked fish under nets was reduced from 12 h to 1 h whilst hand gloves, head covers and nose masks were used during hand-sorting and packaging of fish into sterile zip-lock plastic bags. After applying the GMP based on Table 2 [35] the properties of the new batch of smoked fish was compared to those before the intervention.

**Table 1:** Traditional practices and suggested good manufacturing practices for improving fish quality at two fish smoking facilities in the Cape Coast Municipality.

Observation	Intervention
• Frozen fish was transported to the processing centre without any temperature control.	• Frozen fish was placed in ice-chests containing ice-cubes during transportation to maintain temperature.
• No waste disposal unit was used at the processing centre	• Bins for waste disposal were provided
• Some unit operations, eg. thawing/washing in bowls were done on the bare ground	• Working benches served as platforms for bowls during thawing/washing of fish
• Thaw drips seeped onto the floor, which attracted flies	• Thawing was carried out in containers and drips were collected and properly disposed. Cleaning of processing facilities and down-wash of premises, facilities and equipment, daily sampling for microbial pathogens.
• Fish draining prior to smoking was carried out in the open air.	• Nets were provided to cover fish during draining to keep away insects/flies
• Smoked fish was left in the open during overnight cooling	• Nets were provided to cover smoked fish to keep away insects.
• Safety apparels were not used during fish processing	• Hand sanitizers, hand gloves, hair covers and safety coats were provided for use by the operators.
• Smoked fish was packed into brown paper bags in a basket	• Smoked fish was packed into sterile zip-lock plastics and refrigerated (4–6 °C)

**Table 2:** Analysis of hazards that can occur during traditional smoking of fish and possible good manufacturing practices that can be applied (FAO and WHO, 2020).

Processing step	Potential hazard	What can go wrong?	Interventions that can be applied.
Transportation of frozen fish	Biological	Temperature abuse could increase microbial growth	Transportation of fish in a cold insulated container to main the cold chain.
	Chemical	Histamine may occur due to temperature abuse.	Maintain a cold chain during transportation.
Receipt of fish	Biological	Microbial growth may increase with temperature abuse.	Freeze storage if fish cannot be processed immediately.
	Chemical	Histamine may occur with abuse of temperature.	Freeze storage if fish cannot be processed immediately.
Washing	Biological	Bacteria contamination from the water source.	Potable water source and avoiding multiple use of water.
	Chemical	Chemical and pesticide residue.	Potable water must be used.
	Physical	Dirt in water.	Potable water must be used.
Draining	Biological	Prolonged open air draining can lead to microbial proliferation.	Draining on the warm stove to improve dehydration rate.
	Physical	Contamination from the environment (dust and sand).	Draining under cover nets.
Smoking	Biological	Inadequate heating will not kill all microorganisms	Hot smoking (>80 °C) for 2 h to inactivate microbes.
	Chemical	Polycyclic Aromatic Hydrocarbons.	Reduce excessive smoke by allowing good air circulation in the smoking kiln.
Handling/Packaging	Biological	Recontamination of with microbes.	Observe standard sanitary operation procedures.

### 3.3 Effects of GMP on smoked fish quality

#### 3.3.1 Microbiological quality

The microbiological counts (Log CFU/g) of thawed fish for Facility A, (F_A_) and Facility B, (F_B_) before the intervention were respectively, for the aerobic count (4.18 ± 0.02 and 4.20 ± 0.13), coliform (2.84 ± 0.09 and 2.72 ± 0.09), *E. coli* (1.59 ± 0.07 and 1.11 ± 0.10) and fungi (3.86 ± 0.08 and 3.65 ± 0.07), which could originate from the fish harvesting, handling, storage and packaging stages. The levels were lower than the regulatory limits shown in Table 3 [[36], [37], [38]], depicting that the fish samples were of acceptable microbiological quality. After the hygiene intervention, a decrease in the microbial load was observed (Table 3) probably because of the single use of potable water and the temperature/time combination for the thawing process. The total aerobic count of smoked fish obtained from F_A_ before and after the implementation of the GMP was approximately 3.96 ± 0.12 log CFU/g and 1.52 ± 0.28 log CFU/g, respectively (Table 3). Applying the GMP caused a significant decrease in the aerobic counts of smoked fish obtained from both F_A_ and F_B_. The occurrence of aerobic microbes in the range of 3.95 log CFU/g - 4.98 (log CFU/g) in smoked fish obtained from markets within the Central and Western Regions was reported in other studies [12]. Hasselberg et al. [39] reported a range of aerobic microbial contamination from 4.23 log CFU/g to 6.15 log CFU/g among different species of smoked fish. In a study that compared the occurrence of microbes in smoked fish within the smoking environment to that found in markets, average counts of 4.83 log CFU/g and 5.24 log CFU/g, respectively, was reported [7], showing that the microbial quality of the environment could influence the levels of aerobic microbes on fish. The total microbial load observed in this study was below the acceptable limit of 5.7 log CFU/g set by the Ghana Standard Authority for hot smoked fish [39]. Essentially, the results show that microbial load of smoked fish can be reduced by the implementation of the GMP, which can contribute to the safety and storage quality of smoked fish. Coliform levels of the smoked fish from F_A_ and F_B_ were 1.69 ± 0.12 log CFU/g and 1.74 ± 0.37 log CFU/g, respectively, with *E. coli* levels being 1.15 ± 0.21 log CFU/g and 1.24 ± 0.37 log CFU/g, respectively. No counts (below detection) were observed after implementing the GMP. Some studies on smoked fish have reported the presence of coliforms and *E. coli* [7,12,14,39], which were attributed to post-smoking insanitary handling of the final product. In this study, the absence of both coliforms and *E. coli* from the smoked fish following the GMP implementation could be attributed to the decrease in the time for post-handling of smoked fish and the use of safety apparels along the smoked fish value chain.

**Table 3:** Effects of good manufacturing practices on the microbial quality (Log CFU/g) of smoked fish.

Type of micro-organism	Facility A		Facility B		
	Before GMP	After GMP	Before GMP	After GMP	Regulatory Limit^a^
**Raw fish**					
Aerobic count	4.18 ± 0.02^a^	2.49 ± 0.08^b^	4.20 ± 0.13^a^	2.57 ± 0.04^b^	7.0
Coliforms	2.84 ± 0.09^a^	1.24 ± 0.34^b^	2.72 ± 0.09^a^	1.24 ± 0.09^b^	3.0
*E. coli*	1.59 ± 0.07^a^	1.36 ± 0.08^a^	1.11 ± 0.10^a^	1.55 ± 0.07^a^	2.7
Fungi	3.86 ± 0.08^a^	2.02 ± 0.09^b^	3.65 ± 0.07^a^	2.30 ± 0.17^b^	4.0
**Smoked fish**					
Aerobic count	3.96 ± 0.12^a^	1.52 ± 0.28^b^	4.10 ± 0.28^a^	1.85 ± 0.85^b^	6.0
Coliforms	1.69 ± 0.12^a^	0.00 ± 0.00^b^	1.74 ± 0.37^a^	0.00 ± 0.00^b^	1.6
*E. coli*	1.15 ± 0.21^a^	0.00 ± 0.00^b^	1.24 ± 0.37^a^	0.00 ± 0.00^b^	0.0
Fungi	2.09 ± 0.12^a^	0.45 ± 0.07^b^	2.39 ± 0.12^a^	0.35 ± 0.28^b^	4.0

Arithmetic mean ± SD of duplicate experiments with different superscripts are significantly different at p < 0.05.

a Reference: [Gonzalez-Rodriguez et al. [36], Ryder et al. [37], Health Protection Agency [38], Hasselberg et al. [39].

#### 3.3.2 Physicochemical quality

**Fig. 3:**
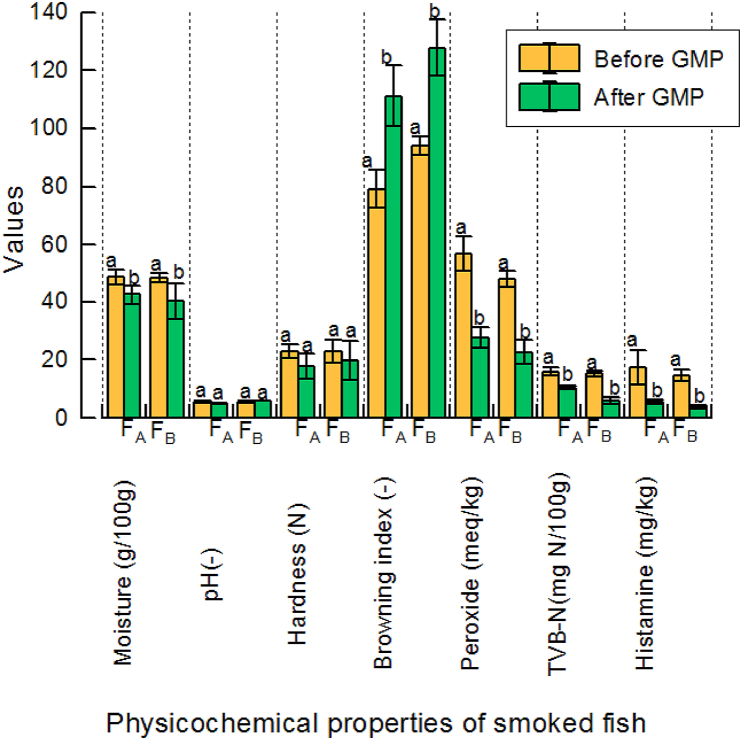
Effects of **good manufacturing practices (GMP)** on the physicochemical quality of smoked fish processed at two local facilities, Facility A (F_A_) and Facility B (F_B_).

Fig. 3 shows that the moisture content of smoked fish was lower after the intervention than before, probably because of adjustments in the fish draining temperature and the quantity of firewood, which improved the continuous supply of heat and hence, moisture loss after smoking. The decrease in moisture could be relevant for the water activity, which could affect the rate of microbial proliferation in fish. The pH and hardness of the smoked fish from F_A_ and F_B_ were in the range of 5.09–5.83 and 17.79 N–23.01 N, respectively (Fig. 3). No significant changes in pH and texture were observed after the implementation of the GMP at the two facilities, however, browning index increased significantly from the range of 79.22 and 94.06 to 111.2 and 127.76, which could be contributed by the increase of the external heating and fish core temperatures. Fish browning is caused by Maillard reaction and the deposition of smoke on the fish [40]. Although the general procedure for fish smoking was comparable among the processors, the operators' source of wood (*Mangifera indica)* for F_A_ and *Petersianthus macrocarpus* for F_B_) could account for the variations in the browning index [40]. The peroxide value of the smoked fish oil decreased significantly after applying the GMP from a range of 48.26–56.85 meq/kg to 22.63–27.65 meq/kg (Fig. 3). Although there was a decrease in the peroxide content after the GMP, the values were still higher than the recommended level of 5 meq/kg WHO [41]. The high peroxide content could be attributed to a limitation in the control of the heat employed during the traditional fish smoking process [42]. Another important indicator of fish quality is TVB-N levels, which estimates the levels of free ammonia and volatile nitrogen compounds, being an indicator of the extent of fish protein decomposition, which could be caused by residual micro-organisms and fish enzymes [43]. The TVB-N levels decreased in F_A_ and F_B_ from 16.0 ± 1.41 mg N/100 g and 15.30 ± 0.86 mg N/100 g to 10.50 ± 0.71 mg N/100 g and 6.0 ± 1.04 mg N/100 g, respectively (Fig. 3), showing a lower level than the acceptable limit of 30–35 mg N/100 g [44]. The decrease could be attributed to the decrease of temperature and time of fish exposure during the fish thawing and draining stages. Histamine, the most commonly reported biogenic amine (BA) in fish, is produced through the decarboxylation of free histidine by the decarboxylase enzyme that is usually released by contaminating microorganisms [34]. The US Food and Drug Administration (FDA) set an acceptable histamine limit of 50 mg/kg [45], whilst the standard for the European Union (EU) is 100 mg/kg [46], showing that the histamine levels recorded in this study, which were in the range of 14.67 ± 1.92 mg/kg to 17.55 ± 5.9 mg/kg, were within the acceptable limits (Table 4) [45,46]. Lower levels of biogenic amines (1.16 ppm–10.36 ppm) from cold-stored fish was reported by Løvdal [10]. The occurrence of histamine on fish is known to be an indicator of poor raw material quality, contamination, and inappropriate storage conditions [47]. Histamine forming bacteria (exemplarily, *Enterobacteriaceae*, *Pseudomonadaceae*, *Klebsiella pneumoniae*) are reported to rapidly grow at warm temperatures [34], and the consumption of biogenic amines-contaminated food could have acute vasoactive, respiratory, gastrointestinal and neurological disorders in humans affecting the general wellbeing [45]. Another important quality index that affects the acceptability of smoked fish from Ghana on the international market is the occurrence of high PAH levels, comprising mainly the PAH4 compounds [48] known to have both carcinogenic and non-carcinogenic effects including skin irritations, child deformities, respiratory disorders, neurological and haematological disorders [49]. In this study, decreases in the levels of individual PAH were observed after the implementation of the GMP. However, the values were well above the recommended levels (Table 4). The EU's maximum limit for benzo[*a*]pyrene and PAH4 (sum of benzo[*a*]pyrene, chrysene, benzo[*a*]anthracene and benzo[*b*]fluoranthene) is set at 2 μg/kg and 12 μg/kg, respectively, for smoked fish products [32], with the minimum values recorded in this study being 14.67 ± 1.92 μg/kg and 17.55 ± 5.90 μg/kg. The high PAH levels of smoked fish has been attributed to the oven type (Chorkor oven) used in the commercial smoking of fish in Ghana [15,16]. PAH contamination of fish is known to be influenced by the type of fuel and the design of the smoking chamber [50]. According to Asamoah et al. [32], the Chorkor oven is the most preferred traditional kiln due to the high product throughput, fuel efficiency, longer lifespan, shorter operation time and lower labour input. Thus, in addition to exploring other types of fire wood, re-engineering of the Chorkor oven to optimize fish contact with the heat source and the smoking time needs to be explored for reducing the PAH levels.

**Table 4:** Effects of the good manufacturing practices (GMP) on the PAH levels of smoked fish.

PAH (μg/kg)	Facility A		Facility B	
	Before **GMP**	After **GMP**	Before **GMP**	After **GMP**
Naphthalene	30.41 ± 3.32^a^	28.17 ± 2.54^a^	26.73 ± 3.72^a^	18.21 ± 1.59^b^
Acenaphthylene	64.78 ± 8.25^a^	43.45 ± 5.40^b^	59.54 ± 8.61^a^	34.16 ± 2.43^b^
Acenaphthene	3.18 ± 0.48^a^	16.67 ± 1.95^b^	3.87 ± 0.35^a^	15.06 ± 2.54^b^
Fluorene	42.69 ± 4.50^a^	27.76 ± 2.80^b^	40.31 ± 5.13^a^	21.29 ± 1.05^b^
Anthracene	43.19 ± 4.79^a^	31.96 ± 3.82^b^	31.82 ± 3.56^a^	31.05 ± 2.12^a^
Phenanthrene	89.05 ± 8.81^a^	68.06 ± 5.38^b^	80.84 ± 10.67^a^	72.96 ± 5.13^a^
Fluorathene	54.02 ± 6.14^a^	32.97 ± 3.33^b^	47.57 ± 4.98^a^	56.64 ± 3.27^b^
Pyrene	45.15 ± 3.94^a^	27.30 ± 2.64^b^	42.92 ± 4.84^a^	38.49 ± 3.01^a^
Benzo(*a*)anthracene	24.25 ± 3.10^a^	15.01 ± 2.61^b^	23.37 ± 2.42^a^	18.87 ± 1.06^b^
Chrysene	38.04 ± 3.15^a^	29.76 ± 2.47^b^	38.53 ± 4.16^a^	33.83 ± 4.17^a^
Benzo(*a*)pyrene	20.13 ± 1.97^a^	14.38 ± 1.04^b^	20.26 ± 2.64^a^	19.24 ± 1.24^a^
Benzo(*b*)fluoranthene	25.64 ± 2.35^a^	16.16 ± 2.41^b^	24.76 ± 2.64^a^	21.34 ± 3.80^a^
Benzo(k)fluoranthene	21.62 ± 2.19^a^	13.22 ± 1.02^b^	19.20 ± 2.57^a^	17.78 ± 2.37^a^
Indenol (1,2,3-c.d) pyrene	15.33 ± 2.13^a^	8.48 ± 1.50^b^	13.13 ± 1.65^a^	12.39 ± 1.14^a^
Dibenzo(a,h) anthracene	3.31 ± 0.25^a^	3.06 ± 0.44^b^	4.16 ± 0.65^a^	3.67 ± 1.69^a^
Benzo(g,h,i) perylene	14.04 ± 0.48^a^	9.17 ± 1.10^b^	15.28 ± 1.93^a^	7.18 ± 0.56^b^
∑PAH	534.83 ± 16.76^a^	385.60 ± 14.63^b^	492.29 ± 13.68^a^	422.13 ± 15.55^b^
∑PAH4	108.06 ± 4.39^a^	75.31 ± 5.41^b^	106.92 ± 8.68^a^	93.27 ± 7.95^a^

Arithmetic mean ± SD of triplicate experiments with different superscripts are significantly different at p < 0.05.

### 3.4 Adoptability of the good manufacturing practices

To assess the adoption level of the intervention, the fish smoking operators were allowed to continue applying the GMP at the studied facilities. After a waiting period of two weeks, an unannounced visit revealed that the operators used only the gloves with the exception of the other interventional measures. Informal discussions with the operators further revealed that the limited enthusiasm to apply the whole set of GMP recommendations was because the fish smoked under the new GMP did not show any competitive advantage in terms of pricing or consumer preferences when compared to that of the other operators who used the unmodified traditional approach. In Ghana, the Food and Drugs Authority (FDA) serves as the national regulatory body for implementing food policies and controlling the production of safe and wholesome food. In addition, several agencies such as the Ministry of Health, Ghana Tourism Board, Ministry of Agriculture, and especially, the local authorities including the Metropolitan Assemblies (Environmental Health and Sanitation Departments) and districts work in tandem with FDA to monitor food safety practices of individuals or groups involved in food production and vending at the micro-small-medium scale [51]. Feglo and Sakyi [52] noted that because of the enormity of resources and time needed to adequately equip the agencies, compared to the urgency to enforce food safety laws, empowering personnel through our educational institution programs to engage in surveillance with rigorous legal backing to issue penalties for noncompliance could help improve the safety and quality of foods. Ababio and Lovatt [51] additionally suggested the need for educating consumers on good hygiene and food safety practices, which could serve as drivers for demanding good quality and safe foods considering that currently, principles of hygiene practices are not legally binding, although laws governing food handling and safety are entrenched in the Ghana Public Health Act 2012 [53].

#### 3.4.1 Limitations of the study

Multiple independent sampling of fish used by the two smoking facilities (F_A_ and F_B_) could strengthen the conclusions derived from the statistical analyses. Future studies to include more smoking facilities and holistic analyses of hygiene indicator micro-organisms could improve the scientific deductions and recommendations for enhancing the safety and quality of the traditionally smoked fish.

## 4 Conclusion

The study revealed that the single use of potable water for fish thawing, draining fish under nets or on warm ovens prior to smoking at an external temperature of 108.1 ± 7.5 °C and fish core temperature of 82.5 ± 3.9 °C for approximately 2 h and packaging in sterile zip-lock bags with minimal exposure to personnel or the environment is helpful for decreasing the occurrence of coliforms and *E. coli* in traditionally smoked fish. By using the GMP, the levels of aerobic microbes and fungi can be significantly reduced to acceptable levels without any significant impact on the physical quality of fish. In addition, the peroxide value and histamine levels can be significantly decreased to acceptable levels. The results show that implementing the basic GMP can help to improve the quality of smoked fish in Ghana. Further studies on a larger population of fish smoking operators and strategies for sustaining the interventions would help to improve the adoption, and thus, processing of smoked fish of improved hygienic and microbiological quality.

## Funding statement

This work was supported by the African Centre of Excellence in Coastal Resilience (ACECoR), University of Cape Coast, Ghana.

## Data availability statement

The data that support the conclusions of this study are available from the corresponding author upon reasonable request.

## CRediT authorship contribution statement

**Vivianne Geraldo:** Writing – original draft, Methodology, Investigation, Formal analysis. **Jerry Ampofo-Asiama:** Writing – review & editing, Writing – original draft, Visualization, Validation, Supervision, Software, Resources, Project administration, Methodology, Investigation, Funding acquisition, Formal analysis, Data curation, Conceptualization. **Cynthia A. Adinortey:** Writing – review & editing, Validation, Supervision, Methodology, Formal analysis, Data curation. **Isaac Okyere:** Writing – review & editing, Resources, Funding acquisition. **Samuel Kofi Tulashie:** Writing – review & editing, Investigation, Funding acquisition. **Alexander Tetteh Kwasi Nuer:** Writing – review & editing, Investigation. **Samuel Bridge Nkansah:** Methodology, Investigation. **Selorm Omega:** Methodology, Investigation. **Salifu Seidu-Larry:** Writing – review & editing. **Nazir Kizzie-Hayford:** Writing – review & editing, Writing – original draft, Visualization, Validation, Supervision, Software, Resources, Project administration, Methodology, Investigation, Funding acquisition, Formal analysis, Data curation, Conceptualization.

## Declaration of competing interest

The authors declare that they have no known competing financial interests or personal relationships that could have appeared to influence the work reported in this paper.
